# Current News and Opinion

**Published:** 1884-11

**Authors:** 


### Current News and Opinion.

### NEW LOCAL ANÆSTHETIC.

At the Ophthalmological Congress held at Heidelberg, during the month of September, a new and most extraordinary local anæsthetic was exhibited—the Muriate of Cocaine. A two per cent solution of this dropped in the eye rendered all the exquisitely sensitive tissues of that organ entirely insensible, so that any operation could be painlessly performed, and a probe could be rubbed over the cornea without causing any unpleasant sensation whatever. The solution caused no irritation, and no subsequent unpleasant symptoms. It is possible that but one side of the story has been revealed, but if the half of what is claimed for the drug is true, it would seem as if it might have a future in dental, as well as ophthalmological practice.

### MOUTH MIRRORS.

Our esteemed friend, Dr. Maynard, of Washington, D. C., has made an improvement in mouth mirrors by substituting pebbles for glass. The doctor finds them superior to the ordinary glass mirrors, as they more clearly reflect the objects they picture. Such mirrors are not in market, but we hope that some of our enterprising manufacturers of dental appliances will get out a supply. As mouth mirrors are indispensable to the dentist, it is important that they be made as nearly perfect as possible. C. E. F.

### ASKINGS AND ANSWERS.

The press of other matter is so great this month, that our question department is entirely crowded out. There are a number of important queries yet unanswered. No. 26 writes a second letter regarding his "trouble," and desires an answer.

"Dentist" (No. 20) also makes a second appeal for information, and desires the opinion of Dr. N. W. Kingsley.

### PLEASE REMIT.

Many of our subscribers have promptly responded to bills recently sent, for which they will please accept thanks.

A few, however, have neglected to remit, and our treasurer would be glad to hear from them.

### ASSISTANT NEEDED.

Wanted, an assistant for a small city in Germany; must be a first-class operator, and well recommended. Apply to

Dr. C. F. W. Bodecker,
60 E. 58th St.

### OBITUARY.

Died—in New York, Sept. 27, 1884, Oliver A. Jarvis, M. D. S.

Dr. Jarvis was born at Islip, Long Island, Aug. 21, 1827. With his father's family he subsequently moved to Ohio. He was a student of Geauga Seminary, and a classmate of the late President Garfield. When twenty-one years of age he went to New York City to study dentistry with Dr. I. Jarvis, his elder brother.

Dr. Jarvis commenced his professional career at a period when opportunities for acquiring a knowledge of a dentist's duties were exceedingly limited, but with a persevering spirit, and by determined effort to properly fit himself for the duties of his calling, he won an honorable position among his fellows. He was decidedly earnest in expressing his views, and was considered one of the prominent working members of the Societies with which he was connected. He was faithful in his endeavors to benefit those who sought his services, and ready to impart to his professional brethren whatever he believed would benefit them. He was prompt to denounce whatever he considered unjust and erroneous, but seemed ever actuated by friendly feelings and generous impulses.

